# Mechanochemical Synthesis of Nanoparticles for Potential Antimicrobial Applications

**DOI:** 10.3390/ma16041460

**Published:** 2023-02-09

**Authors:** Rabindra Dubadi, Songping D. Huang, Mietek Jaroniec

**Affiliations:** Department of Chemistry and Biochemistry, Kent State University, Kent, OH 44242, USA

**Keywords:** porous materials, mechanochemical synthesis, bio-related applications

## Abstract

There is an increased interest in porous materials due to their unique properties such as high surface area, enhanced catalytic properties, and biological applications. Various solvent-based approaches have been already used to synthesize porous materials. However, the use of large volume of solvents, their toxicity, and time-consuming synthesis make this process less effective, at least in terms of principles of green chemistry. Mechanochemical synthesis is one of the effective eco-friendly alternatives to the conventional synthesis. It adopts the efficient mixing of reactants using ball milling without or with a very small volume of solvents, gives smaller size nanoparticles (NPs) and larger surface area, and facilitates their functionalization, which is highly beneficial for antimicrobial applications. A large variety of nanomaterials for different applications have already been synthesized by this method. This review emphasizes the comparison between the solvent-based and mechanochemical methods for the synthesis of mainly inorganic NPs for potential antimicrobial applications, although some metal-organic framework NPs are briefly presented too.

## 1. Introduction

**Figure 1:**
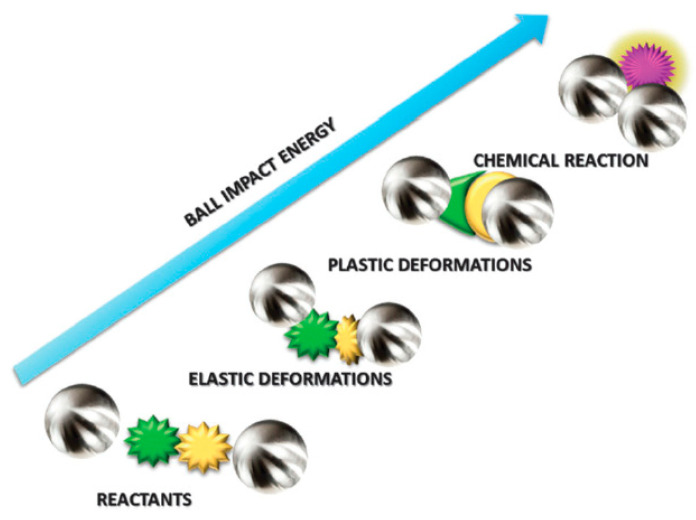
Conversion of reactants to the product in the mechanochemical synthesis. (Reproduced with the permission from Ref. [15]. Copyright © 2015 The Royal Society of Chemistry).

There are two main strategies for the synthesis of nanoparticles (NPs): top-down, and bottom-up [1]. In comparison to the bulk materials, NPs show unique properties such as tunable porosity, controllable particle size, and size-dependent properties, which make them attractive for various applications [2,3,4]. The synthesis of NPs is usually carried out under solvothermal and reductive conditions [5] using conventional heating or ultrasonication [6], microwave irradiation [7], and mechanochemistry [8]. Often, these methods require solvents, and some of them use toxic precursors. Analogous methods are also used for the preparation of NPs with antimicrobial properties [9,10,11,12]. Among them, the mechanochemical synthesis of NPs is an emerging way of performing chemical transformations by means of mechanical forces such as compression, continuous deformation, fractures, shear, or friction [13]. The mechanochemical method involving ball milling has a long history and is still of high importance for the synthesis of various types of materials such as metallics, metal oxides, metal-organic frameworks, organics, carbons, and related nanomaterials. The basic concept for the conversion of reactants to the final product using a mechanochemical strategy is shown in Figure 1 [14,15]. In this process, the reactants accumulate huge potential energy, where shear and friction forces can generate many surface defects, which can substantially improve the reactivity of the final product.

There is a difference between simple grinding and mechanochemical processing. The former simply represents reduction of the particle sizes from bulk to micro- or nano-level dimensions, leading to increased particle activity, while mechanochemistry also involves simultaneous chemical reactions during the grinding process [16]. The grinding process can be categorized into shaker and planetary ball milling. The former is often used for small samples, whereas the latter rotates around the central axis as well as around its own axis. Such motion creates a centrifugal force working against gravity and results in the desired chemical reactions and helps to scale up the product [8].

**Figure 2:**
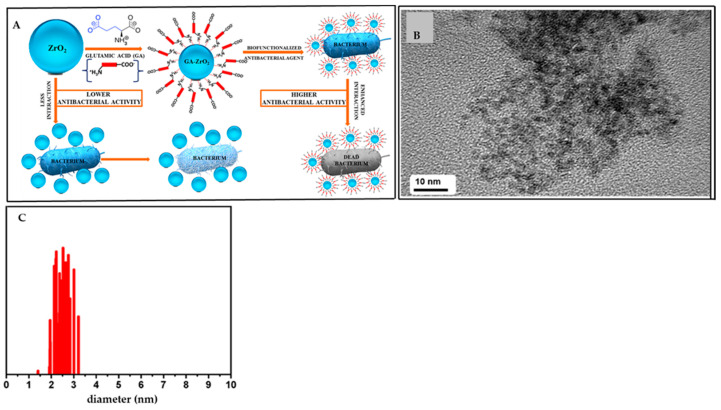
Schematic representation of the synthesis of functionalized ZrO_2_ NPs and their antimicrobial behavior (Panel [**A**]), TEM image of ZrO_2_ NPs (Panel [**B**]), and the corresponding particle size distribution (Panel [**C**]). (Reproduced with the permission from Ref. [28]. Copyright © 2020 American Chemical Society).

In conventional synthesis, solvents play an important role, and often their toxic nature represents the major challenge to be addressed. Mechanochemical synthesis adopts the efficient mixing of reactants using ball milling without or with a very small quantity of solvents. This synthesis is also energy- and time-efficient. Therefore, mechanochemical processing is regarded as an environmentally-friendly (green) synthesis [16]. Mechanochemistry is considered a highly-efficient, easy, and convenient way of synthesizing, modifying, and/or activating nanomaterials, and can easily compete with the conventional synthetic approaches, which often involve multistep processing, a large volume of solvents, risk of byproduct formation, and hazardous chemicals [17,18]. The use of chemical reduction is one of the most common methods for synthesizing different metal NPs such as Ag [19,20], Cu [21,22,23], Au [24], and some composites [25,26]. These are often synthesized by using different toxic and environmentally-hazardous chemicals including NaBH_4_, hydrazine hydrate, formaldehyde, hydroxylamine, hydrogen peroxide, etc. On the other hand, in the mechanochemical process, the solid reagents with large potential energies and strains can create various defects in the final products, and hence the activity of the resulting NPs is highly enhanced [27]. Many studies were already done on the synthesis of various metal and metal-based NPs for antimicrobial applications [28,29,30,31,32]. One of the review articles covers the antimicrobial properties of different NPs synthesized through non-mechanochemical methods [33]. As an example, Figure 2 represents the non-mechanochemical synthesis of ZrO_2_ NPs (Panel A) with possible antimicrobial properties together with a TEM image (Panel B) and particle size distribution (Panel C). On the other hand, one recently-published review article summarizes the mechanochemical aspects of the synthesis of metal oxide NPs but does not cover the antimicrobial aspects of these materials [34]. By contrast, this review article is focused on both mechanochemical synthesis of mainly inorganic NPs and their antimicrobial properties., although some metal-organic framework NPs are briefly presented too.

There are various physical and chemical eco-friendly methods for the synthesis of different nanomaterials such as microwave irradiation, UV-irradiation, sonochemical, mechanochemical, photochemical, and magnetic field-assisted processes [35]. Often, mechanochemical synthesis involves bio-based materials such as lignin with a small amount of metal sources to synthesize metal-containing NPs. This method was also used for the synthesis of Pd, Ru, and Re NPs with lignin as a bio-reducing agent [36]. These green synthetic materials were found to have wide applications ranging from catalysis to biomedical applications [37]. Depending on the shape, size, morphology, and dispersibility of NPs, their utility has been extended in the fields of biological applications. Since the discovery of antibiotics, natural products were effectively used as antimicrobial agents. However, the effectiveness of the available antibiotics has diminished due to the growth of antimicrobial resistance (AMR) and multi-drug-resistant microbes [38].

Antibiotics are chemical compounds that prevent or inhibit the growth of bacterial infections in animals or human beings. Due to the global rise in population, industrialization, change in lifestyle, easy migration, and random or overuse of the available antibiotics, new mutants of bacteria are formed, known as AMR [39]. The WHO has declared AMR one of the top ten global public health threats. AMR occurs when the microorganisms (bacteria, fungi, viruses) change their genetic makeup over time and no longer respond to conventional antibiotics, causing a serious illness or even death [38]. There are many bacterial strains in the world and some of them are growing as a public health threat, as they are multidrug-resistant and cause the deadliest infections—a group of bacteria including *Enterococcus faecium, Staphylococcus aureus, Klebsiella pneumoniae, Acinetobacter baumanii, Pseudomonas aeruginosa,* and *Enterobacter* species, known as ESKAPE bacteria, pose a particularly high threat to humans [40]. These bacteria are growing exponentially worldwide. It is estimated that by 2050 they could cause the death of one person every three seconds. Most of the antibiotics in use these days are the products of the mid-19th century. The situation is getting worse as research on finding new antimicrobial agents has been diverted to the research related to non-communicable diseases [41,42]. The number of recent antibiotics approved or under the pipeline for the approval by FDA is the main attestation of this diversion from antimicrobial research. Now, it is quite late to think critically about novel directions in research toward new antimicrobial drugs to eradicate these superbugs.

A huge investment and technological advancement in the field of biological science set an expectation for the discovery of effective antimicrobial agents. However, this optimistic hypothesis has failed to succeed thus far. There has been only a limited number of drug approvals by the FDA since 2000 [40]. The WHO already warned that if the progress in the development of antimicrobial agents is not sufficiently high, the world is headed towards a post-antibiotic era, where many simple infections will be no longer cured and can result in many deaths [43]. Therefore, the need for significant discoveries of highly effective antimicrobial agents is urgent. Recent research shows that various metal NPs tested for multidrug resistance, along with their possible working mechanisms, are promising. Finding highly effective NPs with strong antimicrobial properties would be one of the milestones in this field. 

## 2. Main Strategies for Synthesis of Nanoparticles

There are many methods, such as sol-gel [44,45], solvothermal/hydrothermal [46], Stöber [47], microemulsion [48], and methods involving microwaves [49], sonochemistry [50] and mechanochemistry, that have been used for the synthesis of NPs. 

Sol-gel is a bottom-up approach, based on a wet chemical synthesis of different NPs (e.g., metal NPs, metal oxide NPs, etc.). During this process, the precursors undergo hydrolysis followed by condensation to give the desired morphology. The final products are obtained after drying. This method has been successfully used to prepare various types of morphologies (nanospheres, nanorods, thin films, etc.) [51].

Stöber method is an effective sol-gel method for the preparation of uniform, homogeneous silica NPs with tailorable pore size and surface functionalities. This method was originated by the ammonia-catalyzed hydrolysis of tetraethylorthosilicate (TEOS) in alcoholic water system [52]. Later, several modifications were made for the preparation of non-silica materials by this approach [53]. 

Solvothermal/hydrothermal methods are very popular for the synthesis of NPs. They refer to the synthesis process carried out in solvents, or in aqueous media in the case of hydrothermal processing. In this process, the chemical reactions occur inside the solvothermal/hydrothermal reactor, known as autoclave [54].

Microemulsions are the stable isotropic mixture of miscible and immiscible liquid phases such as the mixture of oil, water, and different surfactants. In this method, two or more phases are mixed to form microemulsions. During this process nanoscale drops of water remain continuously in an oil phase protected by a surfactant at the interface. The main advantage of microemulsion is controllable drop diameter, which restricts unnecessary interactions with the surrounding allowing formation of NPs with desired particle diameters [53]. There are different microemulsions for this purpose, water in oil (W/O) and oil in water (O/W), and the surfactant as a ternary system are the most common examples. Magnetite [55], iron oxide [56], Au [57], Cu [58], ZnO NPs [59] represent some examples of nanomaterials that can be synthesized by this method.

The use of high intensity ultrasound can produce very high temperature and pressure, which is distinct from other synthetic routes. The irradiation of high energy ultrasound in volatile organic compounds (VOCs) in a nonvolatile solvent result in the dissociation of metal carbonyl bonds and produce the elemental metal atoms. This method can also be used for the synthesis of noble metal nanoparticles, bimetallic core-shell nanoparticles, metal oxides etc. [60]. Similarly, microwave assisted synthesis of various nanoparticles is the next important strategy. This is a simple, fast, easy, and efficient way of synthesizing advanced nanomaterials. Microwaves represent electromagnetic radiation ranging from 300 MHz–30 GHz, which assures an instantaneous and homogeneous heating of the precursor materials. This method can be used for the synthesis of porous nanomaterials such as silica, carbons, metal-organic frameworks, metal oxides etc. [61]. 

In addition to sonochemical and microwave-assisted methods, in which ultrasounds and microwaves are a source of external energy provided to the synthesis mixture, mechanochemistry is one of the oldest methods using mechanochemical forces to reduce the particle size of substances and initiate chemical processes. Due to the advancement of various tools and techniques, mechanochemical grinding/milling becomes one of the popular, safe, and green synthetic tools for the preparation of various types of NPs.

## 3. Mechanochemistry: History and Advantages

The history of mechanochemistry is very long. The first use of the mortar and pestle as a grinding tool can be traced to the stone age. Later, these simple tools were replaced by more sophisticated devices that can be used for the preparation of materials for research and different practical applications. The mechanochemical process involves the chemical transformation of the reactant species by means of various forms of mechanical forces such as compression, shear strain, friction, etc. This process was found to be scripted from 315 BC by Theophrastus in his book, “On Stones” [8]. The working principles of mechanochemistry are still not fully explained, but systematic study was started around the middle of the 19th century and was significantly advanced after the 1960s. The important industrial applications of mechanochemistry include the processing of cement clinker, ores, and powder metallurgy, which adopt fine grinding as a mechanochemical tool and have been used since the 19th century until now [61]. Although the principles and methodologies of mechanochemistry are still being explored, the initial slow progress in this field was accelerated when mechanical alloying emerged. Nowadays, the popularity of mechanochemical synthesis is increasing in various fields, including organic, inorganic, and materials chemistry. Because of the growing popularity of mechanochemistry, IUPAC in 2003 defined mechanochemical reaction as “a chemical reaction that is induced by the direct absorption of mechanical energy” [62].

Mechanochemical synthesis is one of the safest ways to prepare nanomaterials. This synthesis is safer than wet chemical processing. The major advantages of this synthesis are:

(i) Reduction of particle size: ball milling is a physical method that affords the synthesis of particles with reduced sizes down to tens of nanometers.
(ii) Nanostructuring and activation of materials: mechanical grinding can be used for the synthesis of mesoporous materials via template-assisted methods. In addition, mechanochemistry can be applied for the nano-casting synthesis of nanoporous materials [63].
(iii) Doping of nanoparticles: the activity of nanomaterials mainly depends on their surface-to-volume ratio, size, and surface functionality, as well as the active sites present on the surface. The surface properties of NPs can be modified by doping, which is commonly used to enhance their catalytic activity, antimicrobial properties, etc. Moreover, doping permits the realization of desired properties for specific applications such as wastewater treatment, nuclear waste management, and adsorption-based removal of harmful dyes [64,65,66].
(iv) Reduction of reaction time: mechanochemical processing is quicker than conventional synthesis. The reduction of tungsten carbide particles from 2–3 mm sizes to 3 µm takes 70 h in conventional synthesis, whereas the same can be achieved in 3 min in a planetary ball mill [67].
(v) Large-scale production: this method helps to produce high-purity NPs on a large scale [68]. For instance, about 10 g of ternary lanthanum nanoscale coordination polymer was obtained by this solvent-free method [69].
(vi) Low agglomeration: this approach helps to produce the NPs with narrow particle size distribution [70].
(vii) Medicinal value: the use of modern mechanochemistry in the medicinal field as medicinal mechanochemistry expands the scope of this approach [71].

Along with these advantages, some disadvantages of this process are known too. Namely, this method requires high-energy mechanochemical equipment, is prone to particle contamination originating from the container and grinding balls, and it is often difficult to achieve ordered porosity, precise shape, and size due to high energy milling [72,73].

## 4. Mechanochemical Synthesis of Nanoparticles

The basic principle of mechanical synthesis is the grinding of solid materials, which involves the reduction of particle sizes. The essence of mechanochemical processing involves the induction of chemical reactions between raw materials by the input of mechanical energy. This is the most important difference between grinding (top-down approach) and mechanochemical processing [27,74]. The close contact between the milled particles highly enhances the diffusion and chemical reactivity of the reactants [67]. During the ball milling process the plastic deformation, shear stress or shock impact, fracture, and friction due to the collisions induce structural defects and can break chemical bonds. After multiple processes, a new and active state of the material is produced [75].

Mechanochemistry can be used to facilitate reactions at different interfacial systems such as solid-solid, solid-gas, and solid-liquid systems. Specifically, mechanochemical ball milling is extensively used for the synthesis of different types of metallic NPs, metal oxide nanocomposites, and different types of doping processes. There are various types of mills in use for synthesis. Some of them are [75]:

1. SPEX shaker mills
2. Planetary ball mills
3. Attritor mills
4. Modern mills (rod mills, vibrating frame mills)

### 4.1. Synthesis of Metal Nanoparticles

AgNPs were successfully synthesized via mechanochemistry (ball milling) by using lignin as a biodegradable reducing agent without solvents. The synthesized AgNPs showed a very efficient antimicrobial property for both gram-positive and gram-negative bacteria [76]. Some of the studies showed that mechanochemistry can be successfully used for the synthesis of ultrafine Fe, Co, Ni, and Cu NPs [77]. The mechanochemical reduction of binary sulfides of copper, chalcocite (Cu_2_S), and covellite (CuS) by elemental iron resulted in the formation of copper nanoparticles [78]. This semi-industrial approach can also be used in laboratories as well as large-scale production [68,78]. Mechanochemistry helps solve the problems associated with coalescence and oxidation of metallic particles and facilitates particle size reduction by extending the milling time. It also helps to generate the products within a short time, even within a few seconds [79]. The synthesis of AgNPs in the presence of graphite as a reducing agent is the next successful example of ball milling [80]. The latest emerging area of mechanochemistry for the synthesis of nanomaterials is the use of green-type precursors. In this case, mechanochemical processing can be considered bio-mechanochemical synthesis. An example of such processing is the synthesis of AgNPs in the presence of natural products as reducing agents, i.e., *Origanum vulgare* leaf extract [76].

### 4.2. Synthesis of Metal Oxide Nanoparticles

Synthesis of ZnO NPs in an eco-friendly mechanochemical way is based on chemical Reactions (1) and (2). During this process, Zn (OH)_2_ is formed after milling and subsequent heat treatment gives the ZnO NPs [36].

Zn (CH_3_COO)_2_ + NaOH → 2CH_3_COONa + Zn(OH)_2_ (1)

Zn (OH)_2_ → ZnO +H_2_O (2)

There are various routes for the synthesis of metal oxide NPs such as hydrothermal synthesis, chemical bath deposition (CBD), sol-gel method, etc. Most of these syntheses are carried out in the liquid phase and require a large volume of solvents. In contrast, high-energy ball milling converts the bulk materials into fine powder without solvents or with an extremely small volume of solvents. The mechanical energy activates the chemical reagents, which results in producing nanoparticles as the final products [27]. An easy, fast, and green synthetic route for the preparation of different metal oxide NPs makes the mechanochemical process very useful. For instance, the synthesis of Gd_2_O_3_ by mechanochemical processing and subsequent heat treatment was reported [81]. Similarly, other metal oxide nanoparticles including Cr_2_O_3_ [82], ZnO [83,84], ZrO_2_ [85], CeO_2_ [86], SnO_2_ [87], CdO [88], CoO [89], and TiO_2_ [90] were effectively synthesized by this method. 

The biochar (carbonaceous and porous material) exhibits limited adsorption ability to anionic species. For instance, modification of biochar with metal oxide species to form nanocomposites significantly enhances its adsorption capacity. The formation of these nanocomposites by different processes may discharge some contaminants either as a byproduct, or impose contamination risk on the final product. High-energy ball milling can greatly reduce the contamination risk of the final product. It also decreases the particle size and increases the specific surface area and thus introduces plenty of active sites for adsorption. Specifically, combining CuO with biochar can increase the porosity of the resulting composite, enlarge specific surface area, and introduce hydrophilicity, which greatly enhances the adsorption capacity of the composite [91]. The comparison study for the synthesis of some metal and metal oxide NPs through mechanochemical and solvent-based methods is shown in Table 1. It includes the chemicals used, and particle size.

### 4.3. Synthesis of Nanoalloys and Nanocomposites

**Figure 3:**
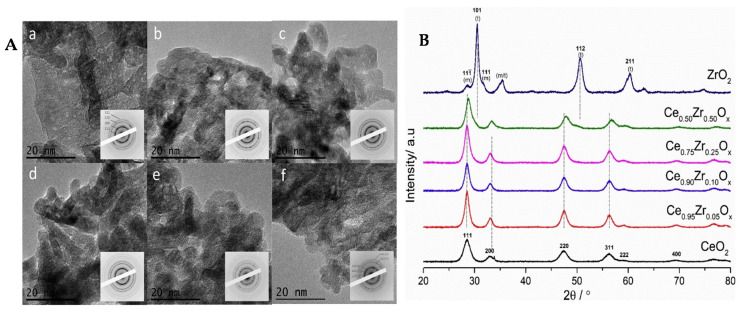
TEM images of (**a**) CeO_2_ NPs; (**b**–**e**) CeZrOx (x denotes the different ratios of Ce:Zr used); (**f**) ZrO_2_; insets are the electron diffraction pattern of the selected areas (Panel [**A**]), and the corresponding XRD pattern (Panel [**B**]). (Reproduced with the permission from Ref. [102]. Copyright © 2019 Elsevier B.V. All rights reserved).

Mechanical alloying is the next advantageous strategy to synthesize mixed metal nanoparticles (alloy nanoparticles). These types of nanoparticles are widely used in catalytic applications as they show some synergetic effects. There are various methods for the preparation of bi- or multi-metallic nanoalloys. Many of the synthetic procedures are analogous to those used for the formation of monometallic NPs. Due to the various technical difficulties and laborious conventional synthetic procedures, mechanochemistry is one of the alternative and easy ways to prepare the metal oxide nanocomposites, supported metal nanoparticles, mesoporous materials, and different coordination polymers because of its simplicity and low cost [100]. For instance, Fe/CaO and Co/CaO nanocomposites were synthesized by inexpensive mechanochemical processing using non-toxic metal oxide precursors [101]. Mechanochemical synthesis can also be used for the synthesis of mixed metal oxide NPs such as ceria-zirconia [102]; a TEM image of such NPs (Panel A) with the corresponding XRD pattern (Panel B) is shown in Figure 3.

### 4.4. Use of Mechanochemistry for Doping and Incorporating Various Species

TiO_2_ has been extensively studied as a photocatalyst and it can be synthesized by different approaches such as sol-gel, hydrothermal, and mechanochemical (ball milling) methods. Doping and co-doping with suitable metallic or nonmetallic elements or coupling with another semiconductor have been used to enhance its properties. Silver-doped TiO_2_ has Schottky defects and behaves as an electron trap. Various methods have already been proposed to dope Ag on the titania surface. However, ball milling is cost-effective, less time-consuming, and an eco-friendly approach. Doped titania nanoparticles are photo-catalytically and biologically active [103]. Another example is a conventional synthesis of porous carbon, which involves a multi-step and expensive process, and produces many wastes as a byproduct. Mechanochemical synthesis of carbons eliminates these shortcomings. Thus, a mechanically-induced self-sustaining reaction can be performed at room temperature to get the N-doped porous carbon or nitrogen-rich carbon materials – specifically, one-pot mechanochemical process involving calcium carbide and cyanuric chloride [104]. Reaction (3) was used to obtain nitrogen-rich carbon material, i.e., C_6_N_3_ carbon nitride:

3CaC_2_ + 2C_3_Cl_3_N_3_ ⟶ 3CaCl_2_ + 2C_6_N_3_ (3)

Similarly, an easy, efficient, and safe method of doping of Mg on hydroxyapatite was achieved by the dry mechanochemical method [105]. Additionally, the mechanochemical synthesis of transition metal-doped ZnO for photocatalytic applications was performed. Co-doping of ZnO highly reduced its photocatalytic activity as the Co ions substituted the Zn ions in the ZnO wurtzite phase. On the other hand, the Mn dopant showed an increased photocatalytic activity at low levels of doping, which was reversed at a higher level of doping [106].

### 4.5. Mechanochemical Synthesis of Highly Porous Nanoparticles

**Figure 4:**
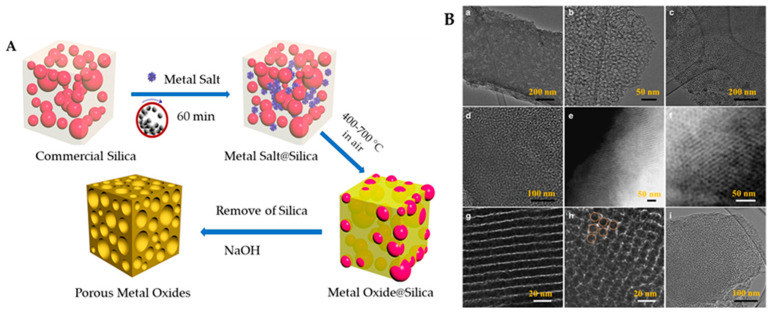
Schematic experimental representation of the mechanochemical nanocasting of porous metal oxides (Panel [**A**]) (reproduced with the permission from Ref. [63], Published Copyright © 2018 American Chemical Society) and TEM images (**a**–**d**) representing different ratios (0.4, 0.6) of Pluronic F127 and tannin to form OMC with different magnifications and STEM-HAADF (high angle angular dark field). Images (**e**–**h**) representing F127: tannin (0.8) where orange circular rings show cylindrical mesopores, (**i**) represents the OMC structure obtained by using P123 Pluronic bock copolymer as a soft template (Panel [**B**]). (Reproduced with the permission from Ref. [109]. Adapted from Pengfei Zhang et al. (2017) under the Creative Commons CC BY license).

Adsorption is an important physical phenomenon that results in attracting atoms or molecules of gas, liquid, or solid phase on the surface. The porosity of material means the presence of various interconnected voids and/or channels in its matrix. IUPAC defines porosity in terms of the size (diameter) of pores and distinguishes three classes of materials: (i) microporous, having pore sizes below 2 nm; (ii) mesoporous, having pores in the range of 2–50 nm; and (iii) macroporous, possessing pores with sizes larger than 50 nm [107]. Microporous materials are further sub-classified as ultra-microporous materials having pore sizes of 0.7 nm or smaller [108]. Mechanochemical synthesis is an emerging method for the preparation of various porous materials [8]. This method overshadows the phenol-formaldehyde polycondensation approach for the formation of porous carbon. The uniform and scalable ordered mesoporous carbons (OMCs) were synthesized using bio polyphenols (tannin), and block-copolymer. This method was modified to incorporate Ni and Zn species into carbons [109]. Figure 4 shows the synthetic route of a mesoporous metal oxide obtained via mechanochemical method (Panel A) and TEM images of carbon synthesized through the solid-state approach (Panel B). Similarly, mesoporous crystalline γ-alumina and modified alumina with a high specific surface area and pore volume were synthesized from boehmite as an alumina precursor via high-energy mechanochemical ball milling [110,111].

**Figure 5:**
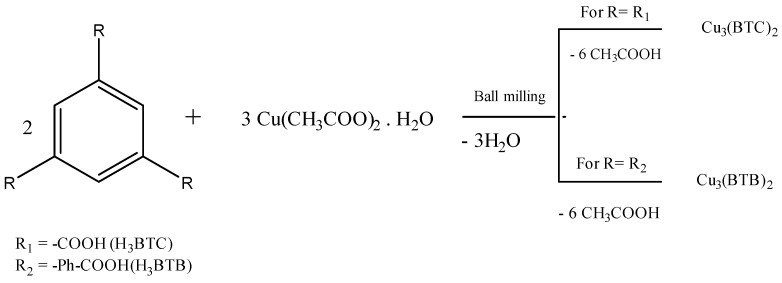
Mechanochemical synthesis of HKUST-1 (copper benzene-1,3,5-tricarboxylate) and MOF-14 {[Cu_3_(BTB)_2_(H_2_O)_3_] (DMF)_9_(H2O)_2_}.

Additionally, ball milling was used to synthesize FeO(OH) nanoflake/graphene and nano Fe_3_O_4_/graphene composites from commercially-available graphite oxide and iron powders [112]. Mechanochemical approach facilitates the synthesis of two- and three-dimensional metal-organic compounds. Figure 5 represents the chemical reaction for the formation of Cu_3_(BTC)_2_ and Cu_3_(BTB)_2_ [113]. A comparison of the mechanochemical activation of metal-organic framework (MOF) (HKUST-1) (S_BET_ = 1713 m^2^/g) with the sample without activation (S_BET_ = 758 m^2^/g), and commercial sample (S_BET_ = 1836 m^2^/g) has been reported elsewhere [113].

## 5. Antimicrobial Applications of Mechanochemically-Synthesized Nanoparticles

High demand for various nanomaterials requires further advancements in optimizing synthetic procedures by using low-cost and renewable precursors, minimizing energy usage, and preparing environmentally-friendly (greener) materials by eliminating toxic chemicals, reducing solvent usage, and avoiding harmful gas emissions by adopting the 12 principles of green chemistry [114]. Mechanochemistry is a promising way to address these issues for the synthesis of different types of nanomaterials. The mechanochemically-synthesized nanoparticles can be successfully used in various areas ranging from adsorption, catalysis, and energy storage to bio-related applications. The advantages of mechanochemistry presented in Section 3 make this method attractive when compared to the conventional synthesis. This section is devoted to NPs with antimicrobial properties, which can easily be synthesized, modified, and activated via mechanochemical treatments, and their comparison with other modes of synthesized particles.

### 5.1. Antimicrobial Properties of Nanoparticles 

**Figure 6:**
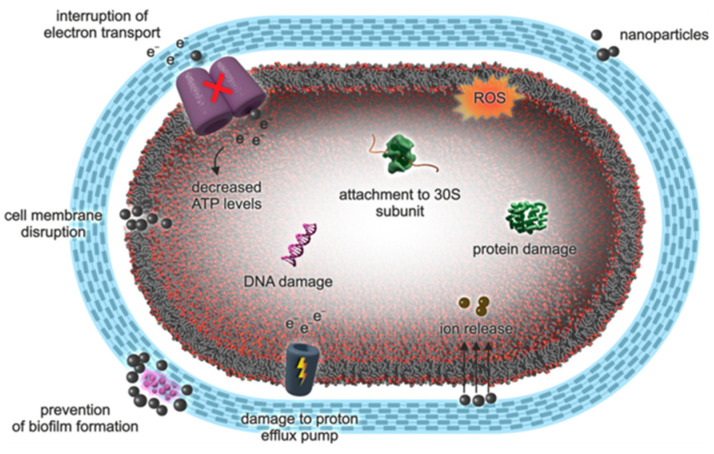
Mechanism of antimicrobial action of nanoparticles. (Reproduced with the permission from Ref. [118]. Adapted from Hochvaldová et al. (2022) under the Creative Commons CC BY license).

Metal-based NPs have been extensively studied in the field of biomedical applications. The antimicrobial properties of materials depend upon various parameters including the nature of the material, size, solubility, and permeability. Similarly, the role of metal-based NPs is unique because they have broad bacterial toxicity (non-specific), and the mechanism is complex and not specific to certain bacterial cells. This might be the reason that bacteria barely develop antimicrobial resistance to these NPs. Hence, the generation of new antimicrobial drugs by using metal-based NPs with adequate antimicrobial activity and low toxicity could be a great accomplishment in the field of biomedicine [115]. There are several nanomaterials that possess antimicrobial properties [116,117]. The general mechanism of the bactericidal effect of NPs is shown in Figure 6 [118].

**Figure 7:**
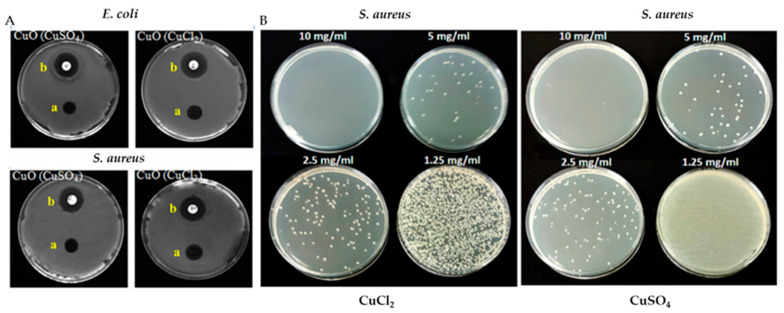
(Panel [**A**]): Determination of ZOI for copper oxide NPs synthesized via mechanochemical method using CuSO_4_·5H_2_O and CuCl_2_·2H_2_O for treating *E. coli* and *S. aureus* bacteria; insets [a] gentamicin antibiotics and [b] copper oxide treated discs in the antimicrobial study. (Panel [**B**]): Determination of MIC and MBC through CFU against *S. aureus*. (Reproduced with permission from Ref. [121]. Copyright © 2019 Elsevier B.V. All rights reserved).

When the size of NPs decreases, their surface-area-to-volume ratio increases and, hence, the bioactivity. There are various theories and mechanisms that explain antimicrobial activities. The main mechanism that explains antimicrobial activity involves the destruction of the cell membrane, interruption of the electron transport chain, generation of reactive oxygen (ROS) species, protein and enzyme disruption, and DNA damage [119,120]. The study of mechanochemical synthesis of NPs with antimicrobial properties is under exploration. Dushkin et al. [115] synthesized a nanocomposite of antibiotic (cephalosporin) with silicon dioxide, exhibiting much higher antibacterial activity than its original counterpart. Similarly, the ultrasmall CuO NPs were synthesized via a mechanochemical method using two different precursors, CuCl_2_·2H_2_O and CuSO_4_·5H_2_O. CuO NPs obtained from the CuCl_2_·2H_2_O precursor showed higher antimicrobial activity, because of their spherical morphology and narrow size distribution [121]. This finding confirms that the smaller particles exhibit higher antimicrobial efficacy. Hence, mechanochemical synthesis generates smaller NPs with enhanced antimicrobial properties. The zone of inhibition (ZOI), minimum inhibitory concentration (MIC), and minimum bactericidal concentration (MBC) determined by colony-forming units (CFU) for CuO NPs derived from both precursors are shown in Figure 7. 

The scope of mechanochemical synthesis is not only limited to the synthesis of metallic or metal oxide NPs but is also widely used in the synthesis of organic, inorganic, and metal-organic framework NPs. For instance, the mechanochemically-synthesized organocatalyst (4-hydroxy-3-thiomethylcoumarin) was used as an antimicrobial agent for pathogenic bacteria and fungi [122]. Similarly, cyclohexanone and indazole derivatives obtained by the mechanochemical method were tested in a wide range of microorganisms, including both gram-positive and gram-negative bacteria, fungi, and yeast. These compounds showed moderate to good antimicrobial properties. The results were compared with standard antibacterial drugs, tetracycline, and the antifungal drug ketoconazole [123]. AgNPs prepared in two different ways, i.e., via conventional green synthesis (using plant extracts) and mechanochemical method, were also compared, and it was found that AgNPs obtained from the conventional method showed better antimicrobial properties. It was further concluded the better antimicrobial properties of AgNPs prepared via conventional green synthesis were due to the unreacted silver precursor remaining in the sample. It seems that the mechanochemical synthesis under optimized conditions could afford better control of antimicrobial properties [124]. The scope of mechanochemically-synthesized NPs, metal oxides, nanocomposites, and MOFs in terms of antimicrobial properties continuous to expand. Table 2 summarizes a variety of metal and metal oxide NPs synthesized via green chemistry (not mechanochemical), together with some basic information and their antimicrobial applications.

The mechanochemical method was used to synthesize various types of oxide nanoparticles, as shown in Table 3. Some of them (not all) were already studied for antimicrobial applications. The antimicrobial properties shown by the metal and metal oxide NPs synthesized through the mechanochemical method are given in Table 3. As a result, the study of biological activity for these NPs could be an area of future research.

**Figure 8:**
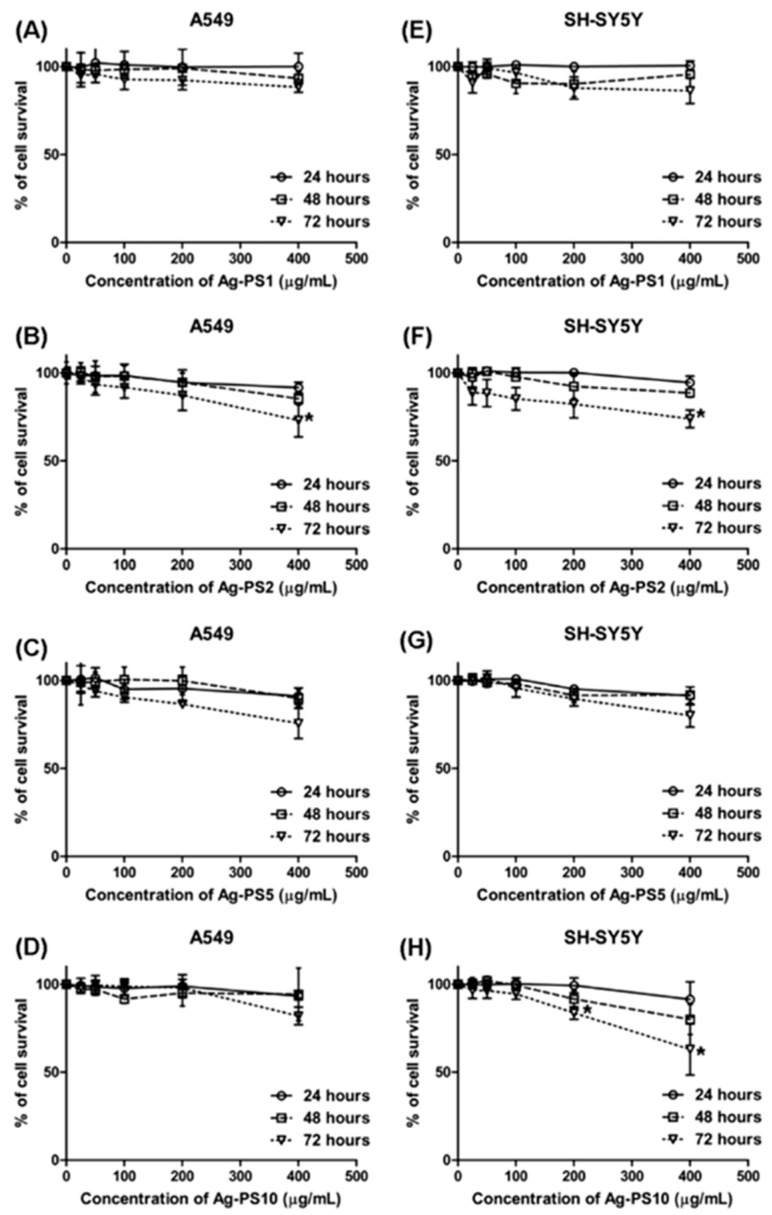
Viability study of MTT [3-(4,5-dimethylthiazolyl-2)2-5-diphenyltetrazolium bromide] assay on A549 (**A**–**D**) and SH-SY5Y (**E**–**H**) human cell lines exposed to various concentrations (0–400 μg/mL) of different Ag-PS NPs for 24, 48 and 72 h; * refers to the significantly different cytotoxic effect than that of the control. (Reproduced with the permission from Ref. [144]. Copyright © 2019 Elsevier B.V. All rights reserved).

The mechanochemical method has also been used for the synthesis of metal complexes. The Co (II), Mn (II), and Fe (II) complexes of amoxicillin were synthesized, and their antimicrobial properties studied. Amoxicillin Fe (II) complex did not show activity against *Staphylococcus aureus* and *Escherichia coli.* The complex with Mn (II) showed the highest antimicrobial activity against *Staphylococcus aureus* in all concentrations [142]. However, the biological activity of these complexes was not compared with the activity of the complexes synthesized by other synthetic methods. It is well-known that ciprofloxacin is a commercial antimicrobial agent. The mechanochemically-synthesized nano-ciprofloxacin showed a significantly increased antimicrobial property [143]. It was found that the bacteriostasis rate of mechanochemically-synthesized nanosized ciprofloxacin is almost twice that of ciprofloxacin powder. Similarly, nanohybrid materials synthesized through mechanochemistry were also studied for their bioapplications. Silver-polysaccharide nanohybrids were synthesized via mechanochemistry and tested for biocompatibility and toxicity. These nanohybrids were found to be biocompatible and less toxic to human cell lines. The viability percentage data of this nanocomposite are shown in Figure 8 **[144]**. The exceptionally low toxicity was expected due to the low solubility of silver precursor from the composite matrix. This study further opens the door toward the mechanochemical synthesis of nanohybrids for biomedical applications.

Copper sulfide prepared by acetate route has been shown to have very high antimicrobial efficacy against gram-negative and to be inactive against gram-positive bacteria. Sulfur-mediated copper sulfide nanocrystals synthesized through a mechanochemical approach showed good antimicrobial activity. CuS with micro-sized particles showed high antimicrobial activity in gram-negative bacteria, whereas the reduction of particle size make them active against both strains of the bacteria [145,146]. AgNPs synthesized from the mechanochemical method using lignin as a reducing agent and polyacrylamide as support were shown to be highly efficient for the complete killing of both gram-positive and gram-negative bacteria strains. They were also effective for multi-drug-resistant strains [147]. Mechanochemistry represents an affordable and sustainable way to synthesize nanoparticles with desired properties. This synthetic strategy can further be enhanced by using “green” precursors such as plant extracts to create materials with antimicrobial properties [145,148].

### 5.2. Porous Materials as Antimicrobial Agents

**Figure 9:**
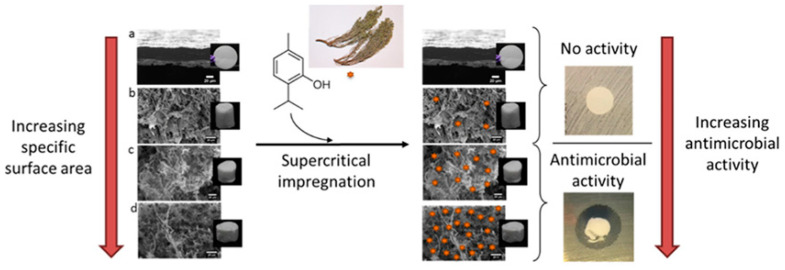
Relationship between the specific surface area and the corresponding antimicrobial properties. (Reproduced with the permission from Ref. [151]. Copyright © 2020 American Chemical Society). (**a**) thymol-impregnated cellulose nanofibrils (CNFs), (**b**) cryogel obtained by freeze-drying the CNF suspension in water, (**c**) cryogel obtained by freeze-drying the CNF suspension in butanol, and (**d**) aerogel obtained by supercritical drying.

There is a wide application of porous nanomaterials in various fields such as adsorption, catalysis, water treatment, sound absorbers, separation, energy storage applications, molecular sieves, etc. In the case of biological applications, metal-based nanoparticles contributed significantly. However, there are some limitations (toxicity, agglomeration, etc.), as a result of which, the effectiveness of these NPs is reduced. The most effective solution to these problems is to immobilize these NPs on various substrates. The commonly-used substrates are porous carbon, graphene, silica beads, etc. [149]. Similarly, mesoporous silica-based materials can be used for bioapplications as the surface functionalization of these materials improves the bioactivity for both in vitro and in vivo study [150]. The impregnation of natural antimicrobial agent Thymol in nanocellulose-based materials under supercritical carbon dioxide conditions was shown to afford material with effective antimicrobial properties. This study further revealed that the cellulose nanofibrils showed better antimicrobial properties because of higher specific surface area [151] (see Figure 9).

The graphene-based materials showed a broad range of antimicrobial properties for bacteria, viruses, and fungi. These graphene-based materials deteriorate the cellular components, mainly proteins, lipids, and nucleic acids. There is a lack of detailed mechanistic study on the antimicrobial properties of graphene-based materials; however, recent research shows that the particle size and morphology, as well as the surface functionalization, lead to creating oxidative stress, cell membrane rupture, and trapping or wrapping [152]. Similarly, carbon nanotubes and fullerenes bind with the lipids and then disrupt the cell membrane and DNA, leading to cell death. Due to the lipophilic properties of fullerenes, which can strongly interact with the membrane lipids, these NPs are biologically more active against gram-positive species [153,154].

### 5.3. Role of Nanoparticles in Antimicrobial Resistance or Multi-Drug Resistance

The awareness of infectious diseases has been highly recognized throughout the world due to the recent finding and outbreak of the COVID-19 (SARS-2) virus. The rate of infection and transmission of this virus has been very difficult to control, and the scenarios caused devastating. The next crisis may be caused by the bacteria-resistant strains that continue to evolve resistance to more and more commercial antibiotics. The generation of antimicrobial resistance is recognized as a global health threat. Research for effective anti-drug-resistant agents is very much necessary. Due to the high surface-to-volume ratio and larger contact with larger numbers of defects, NPs can act as good antimicrobial agents. Additionally, the nanoscale range of these NPs can penetrate the cell membrane and interfere with the biological pathway of the microbes causing apoptosis [120]. The effect of NPs can further be enhanced by reducing their size, conversion into nanoalloys, and functionalization, and can be more effective if combined with existing commercial antibiotics, showing the synergistic effect in enhancing antimicrobial properties [155]. 

These activities of NPs indicate their effectiveness and potential for the next generation of antibiotics [156]. The working mechanisms of NPs as antimicrobial agents are [157]:

(i) Direct exposure to the bacterial cell causing the cell membrane damage;
(ii) Biofilm inhibition;
(iii) Generation of reactive oxygen species (ROS); and
(iv) Disruption of transcription and translation processes.

NPs as antimicrobial agents have been used for a long time. However, the use of these NPs as drug-resistant or multi-drug resistant antimicrobials is not as expected. The in vitro analysis was tested for various metallic NPs such as Au, Ag, Cu, Al, and ZnO against different harmful pathogens, including methicillin-resistant staphylococcus aureus (MRSA), vancomycin-resistant enterococcus (VRE), multidrug-resistant *E. Coli* (MDR *E. Coli*), and MR-ESKAPE [156]. The effectiveness of these NPs to treat most drug-resistant strains is comparable to or better than for existing commercial antibiotics. Studies have shown that if the commercial antibiotics and NPs are merged into hybrid materials, they could be more effective due to the synergistic effect. Additionally, such samples could be prepared in a shorter time, using simple and greener mechanochemical methods, so that their activity could be better than that of their counterparts. Mechanochemical synthesis can afford particles at the nanoscale level, which imparts larger surface area and higher concentration of surface defects. Hence, the implementation of mechanochemical synthesis of NPs as antimicrobial agents or multidrug- or drug-resistant variants could prove a potential field of future research. 

## 6. Conclusions and Perspectives

Synthesis of metal, metal oxide NPs, metal-organic frameworks, doped nanoparticles, multi-metallic alloy nanoparticles, etc. can be achieved by various solvent-based wet chemical methods. All these NPs can be easily synthesized by energy, time, and cost-effective mechanochemistry, which uses a very limited volume of solvents or is solvent-free, is easy, quick, employs eco-friendly chemicals, and activates nanomaterials. This review article mainly covers the mechanochemical synthesis of nanoparticles and their potential applications as antimicrobial agents. Mechanochemically synthesized nanoparticles for antimicrobial applications could be a better option in the field of medicinal science. Therefore, the bio-related research of mechanochemically synthesized materials might prove popular in the future. The particles formed by mechanochemical method possess a lot of defects in the final products, which is advantageous to functionalize and immobilize antimicrobial agents such as metal or metal-based NPs and some existing antibiotics. This property helps enhance effective antimicrobial behavior. The high antimicrobial behavior of mechanochemically synthesized NPs is due to the large surface-area-to-volume ratio, smaller size, and easy functionalization with the existing commercial antibiotics or other metal and metal oxide NPs to form multi-metallic alloy NPs aimed at combating antimicrobial resistance and multidrug resistance. The mechanochemically-synthesized particles could be more active than the conventionally synthesized nanoparticles because of higher surface area and a large number of defects on their surface. Mechanochemistry may afford nanomaterials with better properties in terms of biological, catalytic, and related applications. 

Mechanochemical synthesis follows the principles of green chemistry and is a great strategy to overcome the many drawbacks of wet chemical methods. It might replace the solvent-based strategy and can challenge the conventional synthesis in terms of the effectiveness of the synthesized NPs. Mechanochemistry eliminates or reduces chemical waste and is simple, fast, energy-efficient, and can be scaled up for industrial-scale production. It has been used for the synthesis of various materials for catalysis, adsorption, wastewater treatment, antimicrobial uses, biomedicine, and more. Thus, its use for the synthesis of nanomaterials for different bio-related applications is expected to grow in coming years. The main problem in the biological field is the development of drug-resistant or multidrug-resistant agents due to the overuse or misuse of available antibiotics. The existing nano drugs could be more effective if they are incorporated with the porous nanomaterials. This could be achieved efficiently through a mechanochemical approach. Mechanochemical method could then be a proper way to develop more effective antimicrobial agents, which might be effective against various antimicrobial-resistant and/or multidrug-resistant variants. Some of the previous studies already showed that many metallic and metal oxide nanoparticles have good antimicrobial activities. Consequently, those nanoparticles can be resynthesized or incorporated into various supports such as carbon nanotubes, fullerenes, porous silica, or alumina framework by using mechanochemistry, and they might show improved bactericidal properties due to the synergistic effect to kill a wide range of microorganisms. This approach to nanoparticle synthesis can be extended to various fields such as cosmetic and beauty products, color and paints, toothpaste, and more. As a result, the prospects of mechanochemistry toward development of nanomaterials with antimicrobial and bio-related properties are enormous.

## Figures and Tables

**Table 1:** Comparative study of solvent-based synthesis and mechanochemical synthesis of various NPs.

Solvent-Based Synthesis				Mechanochemical Synthesis	
Samples (NPs)	Size (nm)	Hazardous Chemicals Used	Refs.	Size (nm)	Ref.
Ag	8–50	Hydrazine hydrate, Sodium hypophosphite	[10,92]	39–100	[93]
Au	22 ± 4.6	NaBH_4_	[94]	14.8 ± 6.8	[36]
Cu_2_O	7.5 ± 1.8	NaBH_4_, NaOH	[95]	11	[96]
Fe_2_O_3_	50	H_2_O_2_, N_2_H_4_	[97]	4.21	[13]
ZnO	45–76	Ammonia	[98]	<20 ± 5	[99]

**Table 2:** Antimicrobial properties of various metal and metal oxide NPs.

Sample (NPs)	Synthesis	Size (nm)	Microorganism	ZOI, MIC	Refs.
Al_2_O_3_	Plant Extract, Ultrasonication	96.10, 11–15	*E. coli,* *S. aureus,* *P. aeruginosa*	2.5–10 µg/mL	[125,126]
Ag	Biosynthesis	-	*Proteus,* *E. coli, Bacillus, Pseudomonas*	6–15 mm	[127]
Au	Biosynthesis	53.3	*B. subtilis,* *E. coli,* *K. pneumoniae.*	11.42–17.12 mm	[128]
Cu	Bio reduction	5.3	*E. coli,* *C. albicans.*	Microbial reduction (84–99%)	[129]
Fe_2_O_3_	Biosynthesis	-	*B. subtilis,* *S. aureus,* E. coli, *K. pneumoniae*	10–16 mm	[130]
Fe_3_O_4_	Co-Precipitation		*E. coli,* *B. Subtilis*	6.25 µg/mL	[131]
NiO	Plant Extract	2–21	*E. coli,* *S. aureus*	12 µg/mL 10 µg/mL	[132]
ZnO	Plant Extract	24.5	*K. pneumoniae,* *S. aureus*	9 mm	[133]

**Table 3:** Mechanochemical synthesis of various metal oxide nanoparticles.

Sample (NPs)	Reaction Involved	Milling Time	Size (nm)	Refs.
Bi_2_O_3_	α Bi_2_O_3_ + (ZnO, Fe_2_O_3_, SiO_2_) ⟶ metal oxides doped γ Bi_2_O_3_	5 min–10 h	22.5–67	[134]
CeO_2_	CeCl_3_ + 1.5 CaO + 0.25 O_2_ ⟶ CeO_2_ + 1.5 CaCl_2_	24	19	[135]
Cr_2_O_3_	Na_2_Cr_2_O_7_ +S ⟶ Cr_2_O_3_ + Na_2_SO_4_	-	10–80	[82]
CuO	CuSO_4_·5H_2_O + C_6_H_5_(COOH)(OH) + 3NaOH ⟶ CuO +Na_2_SO_4_ + C_6_H_5_(COONa)(OH) + H_2_ + 7H_2_O	30 min	11.59–22.09	[136]
Fe_2_O_3_	FeCl_3_·6H_2_ + Na_2_CO ⟶ Fe_2_O_3_·6H_2_O + 6NaCl + 3CO_2_	2–5 h	4	[98]
Gd_2_O_3_	GdCl_3_ + 3NaOH + 11 NaCl ⟶ Gd (OH)_2_ + 4NaCl	24	20	[81]
NiO	NiCl_2_·6H_2_O + NaOH ⟶ Ni (OH)_2_ + NaCl	30 min	8–80	[137]
SnO_2_	SnCl_4_ + (NH_4_)_2_CO_3_ ⟶ SnO_2_·H_2_O + NH_4_Cl + 3CO_2_	5 min	3–48	[138]
TiO_2_	TiCl_4_+ (NH_4_)_2_CO_3_ ⟶ TiO_2_·H_2_O + 4NH_4_Cl	5 min	10–50	[139]
ZnO	εZn (OH)_2_ ⟶ ZnO + H_2_O ZnCl_2_ +Na_2_CO_3_ + 6NaCl ⟶ ZnCO_3_ + 8NaCl Zn (CH_3_COO)_2_ + NaOH ⟶ 2CH_3_COONa + Zn (OH)_2_	30 min–6 h	9–36	[36,140]
ZrO_2_	ZrCl_4_ + 2CaO ⟶ ZrO_2_ + 2CaCl_2_	20	8	[141]

## Data Availability

Not applicable.
